# Heritability and Tissue Specificity of Expression Quantitative Trait Loci

**DOI:** 10.1371/journal.pgen.0020172

**Published:** 2006-10-20

**Authors:** Enrico Petretto, Jonathan Mangion, Nicholas J Dickens, Stuart A Cook, Mande K Kumaran, Han Lu, Judith Fischer, Henrike Maatz, Vladimir Kren, Michal Pravenec, Norbert Hubner, Timothy J Aitman

**Affiliations:** 1 Medical Research Council Clinical Sciences Centre, Faculty of Medicine, Imperial College, London, United Kingdom; 2 Max-Delbrück-Center for Molecular Medicine, Berlin-Buch, Germany; 3 Institute of Physiology, Czech Academy of Sciences and Centre for Applied Genomics, Prague, Czech Republic; 4 Institute of Biology and Medical Genetics, Charles University, Prague, Czech Republic; University of Oxford, United Kingdom

## Abstract

Variation in gene expression is heritable and has been mapped to the genome in humans and model organisms as expression quantitative trait loci (eQTLs). We applied integrated genome-wide expression profiling and linkage analysis to the regulation of gene expression in fat, kidney, adrenal, and heart tissues using the BXH/HXB panel of rat recombinant inbred strains. Here, we report the influence of heritability and allelic effect of the quantitative trait locus on detection of *cis-* and *trans*-acting eQTLs and discuss how these factors operate in a tissue-specific context. We identified several hundred major eQTLs in each tissue and found that *cis*-acting eQTLs are highly heritable and easier to detect than *trans*-eQTLs. The proportion of heritable expression traits was similar in all tissues; however, heritability alone was not a reliable predictor of whether an eQTL will be detected. We empirically show how the use of heritability as a filter reduces the ability to discover *trans*-eQTLs, particularly for eQTLs with small effects. Only 3% of *cis-* and *trans*-eQTLs exhibited large allelic effects, explaining more than 40% of the phenotypic variance, suggestive of a highly polygenic control of gene expression. Power calculations indicated that, across tissues, minor differences in genetic effects are expected to have a significant impact on detection of *trans*-eQTLs. *Trans*-eQTLs generally show smaller effects than *cis*-eQTLs and have a higher false discovery rate, particularly in more heterogeneous tissues, suggesting that small biological variability, likely relating to tissue composition, may influence detection of *trans*-eQTLs in this system. We delineate the effects of genetic architecture on variation in gene expression and show the sensitivity of this experimental design to tissue sampling variability in large-scale eQTL studies.

## Synopsis

The combined application of genome-wide expression profiling from microarray experiments with genetic linkage analysis enables the mapping of expression quantitative trait loci (eQTLs), which are primary control points for gene expression across the genome. This approach has been called “genetical genomics”, and recent technological and methodological advances have made its large-scale application feasible in humans and model organisms. Using this approach, the authors have carried out an extensive analysis of the genetic architecture underlying variation in gene expression using a panel of 30 rat recombinant inbred strains. The results are used to explore the relationship between heritability of gene expression, *cis*- and *trans*-acting genetic effects, tissue heterogeneity, and statistical cut-offs of significance, which are important factors for large-scale eQTL studies. By examining large eQTL data from four tissues, the authors provide a detailed picture of *cis*- and *trans*-eQTL features that may help understanding of the genetic regulation of transcription on a genomic scale. The results also show the sensitivity of this approach to discriminate between *cis* and *trans* regulation and the value of the rat system in studying large eQTL datasets from multiple tissues.

## Introduction

Quantitative variation in gene expression levels acts as an intermediate phenotype situated between genomic DNA sequence variation and more complex cellular, organ, or whole body phenotypes. Numerous studies indicate that individual variation in gene expression (i.e., transcript abundance) is heritable in segregating populations [1–5]. Gene expression can therefore be mapped to the genome using standard linkage methods, allowing identification of expression quantitative trait loci (eQTLs), which represent genomic regions for the genetic control of gene expression [6]. This approach has been termed genetical genomics [7,8], and recent technological and methodological advances have made its large-scale application feasible at the level of the genome. One of the most powerful features of this approach is the ability to discriminate between *cis-* and *trans*-acting influences on gene expression and potentially to dissect complex regulatory networks [9,10]. A *cis*-acting eQTL maps to the physical location of the gene itself, whereas a *trans*-acting eQTL maps to a genomic region that is distant from the physical location of the gene being transcribed. By combining the genomic position of the gene encoding each transcript and the position of its eQTL, it is possible to discriminate between *cis-* and *trans-*regulatory control elements of gene expression for thousands of genes across the genome.

A number of genetical genomics studies show that sequence variation in *cis*-acting genes plays a considerable role in determining detectable variability in gene expression, and, accordingly, *cis* effects are usually mapped with high statistical significance [3,11]. *Cis*-acting genes are generally easier to detect by linkage, since they explain a large fraction of the variance of gene expression, and are of great interest as positional candidates for physiological quantitative trait loci (QTLs) [12]. Although *trans*-eQTLs may be associated with lesser statistical significance, they are often detected as clusters, reflecting coordinated regulation of many genes by a single “master regulator” [5]. *Trans*-acting eQTL genes usually explain <20% of the phenotypic variance and may be below a stringent threshold of detection for linkage, since they may reflect genetic regulation that is dispersed across many loci with small effects [13]. Thus, *trans*-regulated genes appear to be more complex (i.e., under polygenic control) than the *cis*-regulated genes and likely reflect the additive outcome of genetic, epigenetic, and environmental regulation [8].

The ability to detect small differences in transcript abundance, or small allelic genetic effects, is partially a function of the power of the study but also relates to the use of corrections for multiple testing of variable stringency that are employed in genome-wide analyses [14]. In most eQTL studies, genome-wide thresholds for significance typically correspond to fold changes (FCs) in gene expression greater than 1.5–2 [15], which may limit the overall sensitivity to detect *cis*-acting effects. When one considers multiple datasets from specific tissues, different magnitudes of genetic effects may affect the relative proportions of detectable *cis-* and *trans*-eQTLs. To date, no large-scale assessment of the relative power to detect *cis*- and *trans*-acting eQTLs in a segregating population and across tissues has been reported. Although thousands of eQTLs are often reported in genetical genomics studies [3,5,11,16,17], the overall distribution of the eQTL genetic effects, heritability, and their relationship with power of eQTL mapping has not been described to our knowledge in any detail.

A central question arising from genetical genomics studies focuses on the relative sensitivity to detect *cis-* and *trans*-acting genetic variation as a function of (i) the heritability of gene expression, (ii) the genetic effect of the eQTL, (iii) the threshold of detection, (iv) the extent of polygenic regulation of gene expression, and (v) how these factors operate in a tissue-specific context [15]. To address these factors, we carried out a large-scale survey of quantitative genetics of transcription by means of integrated linkage analysis and expression profiling to the regulation of gene expression. We carried out this approach in the BXH/HXB panel of rat recombinant inbred (RI) strains, and four tissues were analysed. RI strains were derived by crossing the spontaneously hypertensive rat (SHR) with the normotensive Brown Norway (BN) strain to generate the BXH/HXB panel of 30 RI lines [18], one of the most widely used model systems of human hypertension and metabolic syndrome [18–21]. Along with other features of the RI strains, the ability to make measurements in multiple genetically identical animals from the same strain increases trait heritability, thus facilitating eQTL identification [22,23]. This makes the RI lines an ideal model system to investigate the heritable component of gene expression for *cis-* and *trans*-acting regulators in the context of natural variation in complex physiological processes. Given that in similar study designs [16,17,24] high trait heritability has been used as one of the criteria for statistical significance to map eQTLs, we investigate the effect of heritability filtering on detection of *cis-* and *trans*-acting eQTLs. Here, we show how the overall sensitivity of the genetical genomics approach is determined by factors such as patterns of heritability of gene expression and tissue-specific genetic effects. We discuss how these factors affect the relative power to detect *cis-* and *trans*-eQTLs and highlight the importance of quantifying heritable determinants of gene expression in the context of single tissues.

## Results

### Heritability of Gene Expression in RI Strains

Gene expression profiles and trait heritability (*h* ^2^_trait_) across 30 RI strains were investigated in fat, kidney, adrenal, and left ventricle (LV) tissue for each transcript considered in this study (see Materials and Methods). The median (25%–75% quartiles) *h* ^2^_trait_ is 0.14 (0.11–0.18) for LV, 0.17 (0.13–0.22) for fat, 0.14 (0.10–0.20) for kidney, and 0.17 (0.12–0.24) for adrenal. We observed that the distribution of heritable gene expression is comparable across tissues (*p* > 0.05, chi-square test; Figure S1), suggesting a similar extent of the genetic component segregating in the RI strains. The estimates of *h* ^2^_trait_ were positively correlated with the average levels of expression (Spearman's rank correlations: 0.33 in LV, 0.47 in fat, 0.1 in kidney, and 0.4 in adrenal, *p* < 0.05 in all tissues). We calculated the number of genes exhibiting different *h* ^2^_trait_ and observed no significant difference in the proportions of heritable transcripts across four tissues (Figure S2).

### Global Analysis of Heritability and Allelic Effect of eQTLs in Four Tissues

Table 1 summarises the mapping results in four tissues for the major eQTLs (i.e., the eQTL with the highest statistical significance for each probeset) detected in *cis* or *trans* for each transcript. The median for *h* ^2^_trait_, QTL effects, and heritability of the eQTLs (*h* ^2^_QTL_) are also reported for each threshold of significance. For all major eQTLs detected at *p* = 0.05, the median *h* ^2^_trait_ ranges from a minimum of 0.14 for the *trans*-eQTLs to a maximum of 0.37 for the *cis*-eQTLs. At higher levels of significance the *h* ^2^_trait_ increases for both *cis-* and *trans*-acting eQTLs, but it is consistently lower for the *trans*-eQTLs (Table 1).

**Table 1:**
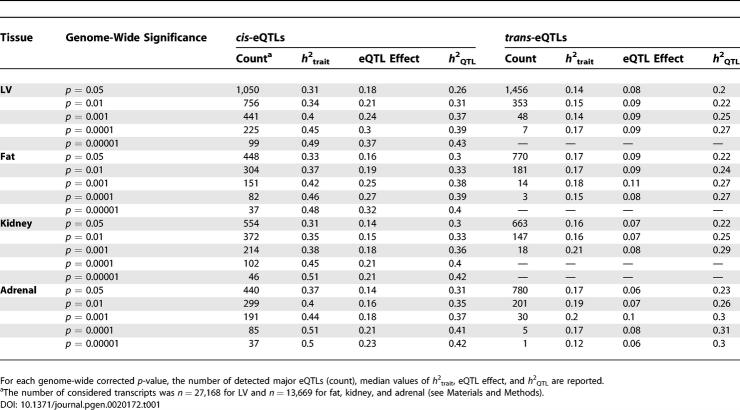
*Cis-* and *Trans-*eQTLs Detected in LV, Fat, Kidney, and Adrenal Datasets at Different Genome-Wide Thresholds of Significance

**Figure 1:**
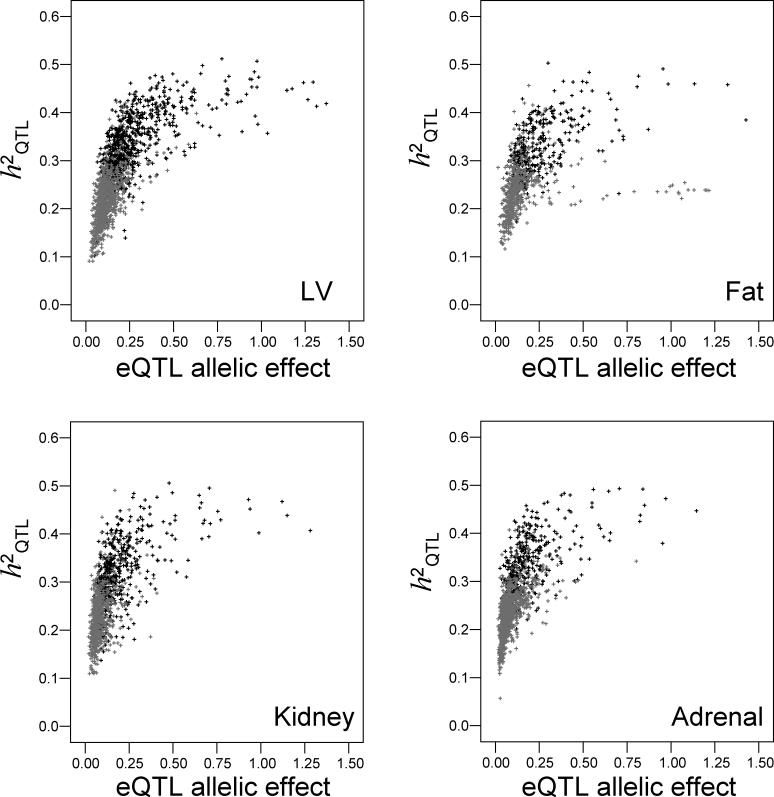
Genetic Architecture of Genetic Variation in Gene Expression For each considered transcript the major eQTL was identified by linkage analysis (genome-wide significance, *p* = 0.05) and characterised as *cis* or *trans*. Additive allelic effect and heritability (*h* ^2^_QTL_) for each *cis-*eQTL (black symbol) and *trans-*eQTL (grey symbol) were plotted for LV, fat, kidney, and adrenal tissues.

At genome-wide significance (*p* = 0.05) eQTL effects are relatively small, ranging from 0.06 (*trans*-eQTLs) to 0.18 (*cis*-eQTLs). Median allelic effects increase at lower *p*-values up to 0.37 for *cis*-acting eQTLs and 0.11 for *trans*-acting eQTLs. The distribution of allelic effects in the range 0–1.5 (i.e., absolute FC in the range 1–8) and relative *h* ^2^_QTL_ for all major eQTLs mapped with genome-wide significance is reported in Figure 1. The vast majority of the eQTLs explained less than 40% of the genetic variance, suggesting that undetected eQTLs account for the rest of the variance in such transcripts. Notably, the strongest eQTLs explained >30% of genetic variance for ~26% of the transcripts, whereas only ~3% of both of *cis*- and *trans*-acting eQTLs (detected at high significance levels, *p* < 10^−4^) exhibited considerably large QTL effects and *h* ^2^_QTL_ > 0.4.

### Heritability of Gene Expression for Detection of *Cis-* and *Trans-*eQTLs

We investigated the *h* ^2^_trait_ as a predictor of the existence of all significant eQTLs in LV, fat, kidney, and adrenal tissue, using receiver operating characteristic (ROC) curves (Figure S3). For the eQTLs detected at *p* = 0.05, the maximum area under the ROC curve (AUC) is 0.73 (standard error [SE] 0.007), which is commonly associated with inaccurate prediction [25]. Heritability appears to be a better predictor (minimum AUC in all tissues = 0.88 [SE 0.011], average AUC across tissues = 0.91) only for the eQTLs detected at *p* = 10^−3^ (Table S1). The ability of the *h* ^2^_trait_ to predict the existence of an eQTL improves by increasing the cut-off of significance for linkage, and at *p* = 10^−5^ the AUC is greater than 0.95 in all tissues (Figure S3; Table S1). We then considered the ability of heritability to predict the existence of *cis-* or *trans*-eQTLs separately. In all tissues, ROC analysis showed that *h* ^2^_trait_ is a poor predictor of the *trans*-eQTLs detected at *p* = 10^−2^: AUC ranges from 0.47 to 0.53. At each threshold of significance, the null hypothesis that the AUC equals 0.5 cannot be rejected for the *trans*-eQTLs. As such, the *h* ^2^_trait_ is no better than chance in predicting a detectable *trans*-eQTL. For the *cis*-eQTLs, we observed higher accuracy in the prediction: AUC ranges from 0.69 (eQTLs detected at *p* = 10^−2^) to 0.77 (eQTLs detected at *p* = 10^−5^), and the AUC is significantly different from 0.5 at each level of significance.

**Figure 2:**
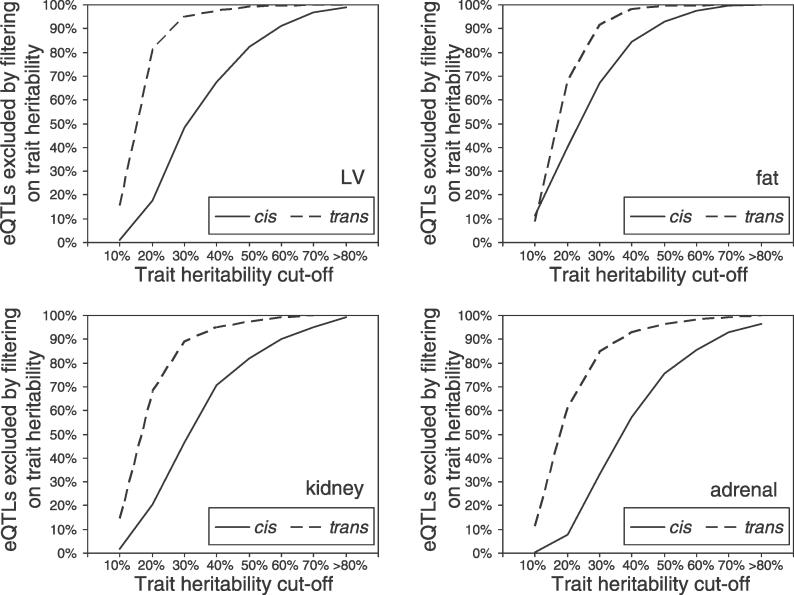
Proportion of eQTLs That Are Excluded when Transcripts are Filtered Based on *h* ^2^_trait_ Percentages were calculated as follows: (1 − (eQTL_TOT_ − eQTL_*h*^2^__cut-off_)/eQTL_TOT_) × 100, where eQTL_TOT_ is the total number of eQTLs detected at *p* = 0.05 without filtering based on *h* ^2^_trait_ (see Table 1), and eQTL_*h*^2^__cut-off_ is the total number of eQTLs observed when transcripts were filtered based on a given trait heritability cut-off. For any trait heritability cut-off, the number of excluded eQTLs is higher for *trans*-acting eQTLs, since they are usually detected for transcripts with low heritability of gene expression.

When high trait heritability was used as the criterion to identify transcripts for which genetic linkage is expected to be more reliable, a significant proportion of eQTLs could not be detected. Given the actual number of eQTLs mapped with genome-wide significance in this study, we calculated the percentage of *cis-* and *trans*-eQTLs that will be discarded when various *h* ^2^_trait_ cut-offs are used (Figure 2). For example, if *h* ^2^_trait_ = 20% is accepted as a prerequisite to map an eQTL and hence eQTLs called from transcripts showing *h* ^2^_trait_ < 20% are not considered, 65% to 80% of the *trans*-eQTLs and 10% to 40% of the *cis*-eQTLs will be excluded from the eQTL dataset (Figure 2).

### Power to Detect *Cis* and *Trans* Effects and False Discovery Rate

**Figure 3:**
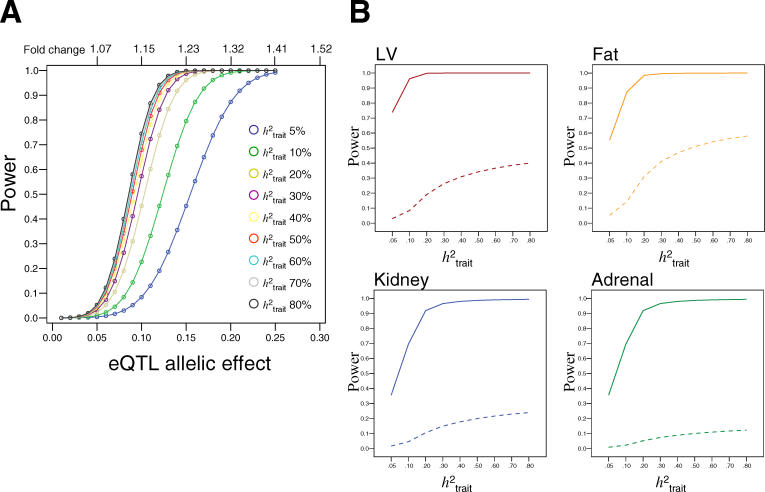
Power to Detect a Major eQTL in the RI Strains (A) Statistical power to detect an eQTL of given allelic effect is shown for various estimates of heritability of gene expression in 30 RI strains with four biological replicates. The eQTL allelic effect is an estimate of the absolute change in the transcript abundance that would be produced by substituting a single allele of one type with that of another type in the population (see Materials and Methods). Absolute FCs, corresponding to the allelic effects on the primary *x*-axis, are also reported on the secondary *x*-axis. (B) Statistical power to detect *cis-*eQTLs (solid line) and *trans-*eQTLs (dashed line) (detected with genome-wide significance, *p* = 0.05) is shown for various heritabilities of gene expression (*h* ^2^_trait_) in LV, fat, kidney, and adrenal tissues. Specific allelic effects for *cis-* and *trans*-eQTLs were defined in accordance with those observed in this study at *p* = 0.05 (reported in Table 1).

Figure 3A shows the power for the minimum detectable effect at different *h* ^2^_trait_ values when allelic effects are in the range 0–0.30 (i.e., absolute FC between one and 1.52). For effects greater than 0.25, the power tends to 100% for all considered values of *h* ^2^_trait_. Small effects are detected with low power, and when the *h* ^2^_trait_ is 0.8–0.9, we achieve ~75% power for an effect of 0.10, which is equivalent to an absolute FC of 1.15. For lower heritabilities of the trait (*h* ^2^_trait_ = 0.2–0.3), the minimum detectable eQTL effect is higher, ~0.12 (i.e., an absolute FC of 1.18). The power to detect small effects increases with the number of biological replicates within each strain, with a significant improvement observed in the range of two to six replicates (Figure S4), and with the number of RI lines (Figure S5). Similar analyses showed that the power increases for eQTLs mapped at higher significance levels (unpublished data), indicating that stronger effects are associated with lower *p*-values.

Given the effects for *cis-* and *trans*-eQTLs detected with genome-wide significance in this study (Table 1), we derived the expected power for the *cis* and *trans* allelic effects in the LV, fat, kidney, and adrenal tissue. Figure 3B shows that the maximum expected power to detect average effects of *trans*-eQTLs is low in all tissues, ranging from 12% to 60% (at high *h* ^2^_trait_). In contrast, the *cis*-regulated genes are expected to be detectable with high power across all tissues. As indicated by tissue-specific allelic effects of the *trans*-eQTL, we expect different power for *trans*-regulated genes across tissues (Figure 3B).

**Figure 4:**
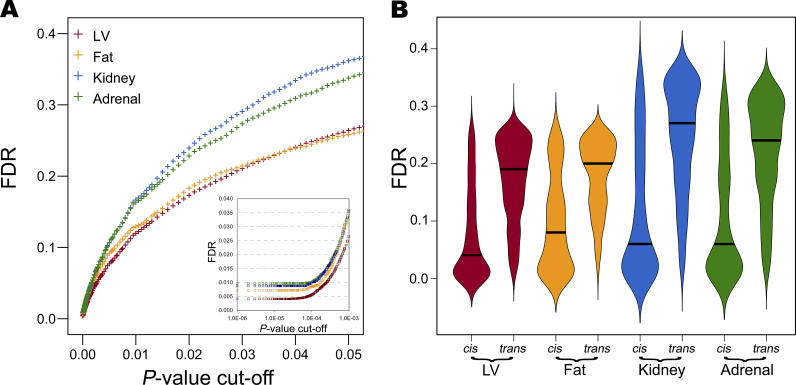
FDR in LV, Fat, Kidney, and Adrenal Tissues (A) For each major eQTL detected in LV, fat, kidney, and adrenal tissue, the expected FDR was calculated and plotted against different *p*-value thresholds in the range 10^−6^–0.05. Insert: FDR for various *p*-values in the range 10^−6^–10^−3^. (B) Vase box-plots for the FDRs of the *cis-* and *trans*-acting eQTLs detected at *p* = 0.05 in LV, fat, kidney, and adrenal tissue. Vase box-plots are box-plots where the width of the box at each point is proportional to the density of the data there. The thick line indicates the median FDR for each distribution.

The lower power to detect *trans*-eQTLs at *p* = 0.05 in kidney and adrenal is reflected in the tissue-specific false discovery rate (FDR) (Figure 4A). At *p* = 0.05, we observe an FDR of ~35% in kidney and adrenal, whereas the FDR is only 26% in LV and fat. This difference in FDR is noticeable for a *p*-value cut-off of >10^−3^, and below this threshold all linkage datasets show similar FDR patterns (Figure 4A, insert). Given that *trans*-acting eQTLs are normally detected at higher *p*-value thresholds, they show higher FDR levels than *cis*-regulated genes (Figure 4B). Across all tissues, at *p* = 0.05 the median FDR ranges from 4% to 8% for the *cis*-eQTLs and from 19% to 27% for the *trans*-eQTLs. For *trans*-acting eQTLs, kidney and adrenal show higher FDR levels (median 24%–27%) than LV and fat tissues (median FDR 19%–20%).

## Discussion

We investigated the overall sensitivity of the genetical genomics approach to discriminate between *cis* and *trans* regulation within and between tissues. Our analysis was carried out in the BXH/HXB panel of rat RI strains and in four tissues: LV, fat, kidney, and adrenal, from which expression profiles were generated. RI strains are a suitable genetic system for global analysis of heritable patterns of gene expression, allowing direct estimation of genetic and environmental components of phenotypic variance [22]. We examined how *cis* and *trans* genetic factors contributed to the global heritability of gene expression observed across tissues. Quantifying the extent of such contributions is of great importance to understand how genetic influences of gene expression are structured within the population and may occur unevenly in the context of specific tissues [26].

We provided evidence for a significant heritable component of quantitative variation of gene expression in all tissues. On the whole, at least 20% of the transcripts showed *h* ^2^_trait_ > 20%, and the overall proportion of heritable genes was similar across all tissues (Figure S2). The value of *h* ^2^_trait_ is higher for those transcripts that were mapped to *cis*-acting eQTLs (31%–51%) than *trans*-eQTLs (12%–21%), thus suggesting that larger differences in transcript abundance are associated with *cis*-acting genetic variation in gene expression (Table 1). Although *h* ^2^_trait_ is positively correlated with the average levels of expression, the proportions of heritable transcripts do not vary by the levels of expression observed in different tissues (unpublished data). We also observed that amount of variation in gene expression levels is consistent across tissues, indicating that the estimates of heritability are not significantly influenced by intra-specific variability between tissues (see Materials and Methods).

It is commonly accepted that the power and precision of genetic mapping will be significantly affected by the magnitude of trait heritability [27]. In practice, the larger the environmental variance (i.e., lower *h* ^2^_trait_), the less likely an eQTL will be detected. However, detection of a significant eQTL does not appear to be entirely determined by the sole estimate of *h* ^2^_trait_. We showed that in large-scale analysis of gene expression phenotypes, *h* ^2^_trait_ by itself is not a consistent predictor of the eQTLs, and its efficiency depends considerably on the *p*-value thresholds at which eQTLs are mapped (Figure S3; Table S1). In addition, we have shown that at each level of significance of the eQTL, *h* ^2^_trait_ is an unreliable parameter in predicting putative *trans*-eQTLs. When *h* ^2^_trait_ is used as a filter to improve detection of linkages [16,17,24], a significant proportion of eQTLs will be discarded from the study. For example, we have shown that conservative filtering approaches (*h* ^2^_trait_ > 20%) result in a significant decrease (65%–80%) in the *trans*-acting eQTLs detected with genome-wide significance (Figure 2). This could have important consequences for identification of clusters of *trans*-acting eQTLs, or *trans*-eQTL “hotspots”, which may represent common regulation by a single gene. It should also be noted that the relative proportion of *trans*-acting eQTLs tends to drop as the statistical significance for eQTL detection is increased (Table 1). This suggests that using conservative filtering approaches may result in many false negatives and in fewer *trans*-eQTL “hotspots” being identified, biasing detection towards *cis*-acting eQTLs.

The relationship between *h* ^2^_trait_ and different detection of *cis-* and *trans*-eQTLs may be a result of their specific mono-, oligo-, or polygenic influences on gene expression. Because sequence variations within single *cis*-acting genes may have a substantial impact on transcription, the measured heritability of gene expression can be very high, indicating existence of major *cis*-regulated eQTLs that explain most of the phenotypic variance [13]. This argument may account for the better efficiency of *h* ^2^_trait_ in predicting highly significant *cis*-eQTLs, since most of them are expected to be inherited essentially as monogenic traits. We found that whilst *cis*-eQTLs have substantial *h* ^2^_QTL_ (26%–31%), the median *h* ^2^_QTL_ observed for *trans*-eQTLs mapped at *p* = 0.05 is only 20%–23%. However, at higher levels of significance we observed more robust *h* ^2^_QTL_ values for both *cis* and *trans* loci (Table 1). This can be indicative of an over-estimate of the eQTL effect size due to the relatively small sample size (Beavis effect [28]) or of a combined effect of a number of physically linked QTLs of small effect [27]. Importantly, we detected only a small proportion of eQTLs (mostly *cis*-acting) that exhibit big allelic effects and account for more than 40% of the phenotypic variance. These data suggest that global variation in gene expression is also likely to be under the influence of multiple loci with small effect alongside a major *cis*-eQTL. This explanation is consistent with previous data in yeast, where only 3% of highly heritable transcripts are explained by single-locus (i.e., monogenic) inheritance and 50% are consistent with more than five controlling loci of equal effect [29,30].

We and others [5] detected a substantial relative proportion (54%–64%) of *trans*-acting eQTLs at *p* = 0.05, and this percentage decreases at higher levels of significance [11]. At each cut-off for linkage, the median effect observed for *trans*-eQTLs (0.06–0.11, FC 1.09–1.16) is well below that observed for the *cis-*eQTLs (0.14–0.37, FC 1.21–1.67). This suggests that detection of *cis*-eQTLs will be facilitated because of their larger effects and that, on a large scale, this study design is more sensitive for detection of *cis* than *trans* effects. We showed that when *h* ^2^_trait_ is greater than 10%, the expected power to detect major *cis*-eQTLs is greater than 70% in all tissues. In contrast, detection of *trans*-eQTLs is limited by low statistical power (12%–60%), as a result of their generally smaller genetic effects. For the eQTLs detected at *p* = 0.05, we observed tissue-specific differences in the allelic effects of both *cis-* and *trans*-eQTLs. In particular, kidney and adrenal tissues showed enrichment of *trans*-eQTLs with smaller effects (Figure S6). Although these differences are negligible for detection of *cis-*eQTLs*,* they appear to be significant for detection of *trans*-eQTLs. We found that, when the major eQTL is *trans*-acting, minor differences in allelic effects are expected to have a significant influence on the statistical power to detect *trans*-eQTLs (Figure 3B). As a result, at genome-wide significance, where the majority of eQTLs are regulated in *trans,* kidney and adrenal showed a higher proportion of false positives among the detected eQTLs (FDR 34%–36%) than that observed in LV and fat tissues (FDR ~26%). Our data indicate that *trans*-eQTLs account for most of the high FDR expected at *p* = 0.05 (Figure 4B), and this goes together with the difference in the statistical power for *cis-* or *trans*-eQTLs across tissues. This observation has a number of possible explanations. First, technical variability in microarray experiments (e.g., due to quality of tissue samples or inherent homogeneity of hybridization [31]) may account for the different sensitivity to *trans*-eQTLs across experiments. However, if this is the case, we should not observe a similar extent of variability and *h* ^2^_trait_ consistent in all tissues. Therefore, we can exclude technical variation as the primary or sole cause of different sensitivity to *trans*-eQTL effects. Second, actual differences in tissue composition may influence the ability and power to detect *trans*-eQTLs by linkage and the FDR in this study design. It is plausible that the increase of biological variability in more complex and heterogeneous tissues—such as adrenal and kidney—or susceptibility to transcription variation in response to environmental stimuli could explain the observed distribution of *trans*-eQTL effects.

Although the patterns of heritable gene expression were similar across tissues, the fat eQTL dataset showed a set of 20 *trans*-regulated genes with high allelic effect (>0.30) but low heritability of the eQTL (*h* ^2^_QTL_ ~ 0.2; Figure 1). Inspection of the biological function of these genes revealed that they are all highly expressed in skeletal muscle belonging to the sarcomeric skeletal muscle contractile apparatus. Because the peritoneal fat pad sits in close proximity to the retroperitoneal muscle, and there was marked, but highly correlated, intra-strain variability in raw expression values of this set of genes (i.e., low *h* ^2^_QTL_), we consider it likely that this cluster of apparent *trans*-eQTL effects is due to contamination of the fat pad samples with small quantities of skeletal muscle. Apart from this cluster of skeletal muscle genes, there are very few if any *trans*-eQTL genes from fat or other tissues showing a similar pattern of low *h* ^2^_QTL_ and high eQTL allelic effect. This finding suggests that this possibly confounding effect is limited to this cluster of genes and does not have a significant bearing on the other *trans* linkages identified in our studies. However, it shows the sensitivity of this experimental design to tissue sampling variability, and the value of this type of analysis for demonstrating the integrity of eQTL datasets.

In summary, this is to our knowledge the first report to address the relative sensitivity to detect *cis-* and *trans*-eQTL effects based on the observed patterns of heritable gene expression across multiple tissues and that takes into account their thresholds of detection and FDR. *Trans*-regulated genes showed small genetic effects that affect the power to detect these eQTLs in different tissues. Although variability in gene expression across tissues is well known [31], we believe that the higher FDR levels for *trans*-eQTL genes in adrenal and kidney may be correlated to high cellular heterogeneity of those tissues, compared to LV or fat. Importantly, tissue-specific features of the *trans*-regulated genes are not explained by either observed patterns of heritable gene expression or variability of expression levels. When major-effect eQTLs are mapped in *trans,* their detection may be precluded by overly stringent statistical cut-offs of significance or by adopting conservative filtering approaches based on trait heritability. This is likely to occur to different extents in different tissues, and we showed that it is a function of the allelic effects of *trans*-eQTLs. Thus, integration of data from multiple sources requires careful consideration and may, in some cases, not be appropriate. For *cis*-eQTLs, detection is enhanced by their bigger effects, and the identification of shared *cis*-acting regulators of gene expression between tissues is facilitated. Because of their more complex and polygenic nature, the profiles of *trans*-acting eQTLs are likely due to tissue-specific regulation, thus limiting the detectable shared *trans*-eQTLs across experiments [3,11,16]. By examining genome-wide expression and linkage data from four tissues, we provide here a detailed picture of *cis*- and *trans*-eQTL features that may help in understanding the genetic regulation of transcription. This study highlights that large-scale identification of *trans*-acting loci can be strongly influenced by tissue-specific factors, which are likely to be key determinants in the feasibility of discovering regulatory gene networks at the level of the organism.

## Materials and Methods

### RI strains and tissues.

The set of 30 RI strains used in this study was produced by inbreeding between members of the F2 generation resulting from the cross of the two highly inbred strains BN.Lx/Cub and SHR/Ola [18], designated here as BN and SHR. Rats were housed in an air-conditioned animal facility and allowed free access to standard laboratory chow and water. All experiments were performed in agreement with the Animal Protection Law of the Czech Republic (311/1997) and were approved by the Ethics Committee of the Institute of Physiology, Czech Academy of Sciences, Prague, Czech Republic. Animals were killed at 6 wk of age. Tissues from four unfasted males of each RI strain were harvested between 9 and 10 a.m., immediately removed, frozen in liquid nitrogen, and stored at −80 °C.

### Microarray analysis.

The original experimental design is discussed in Hubner et al. [11]. In addition to the fat and kidney data described, we also analysed adrenal and LV data (T. J. Aitman, M. Pravenec, N. Hubner, and S. A. Cook, unpublished data) from the same panel of RI strains and experimental design. Gene expression summary values for Affymetrix GeneChip data (Affymetrix, Santa Clara, California, United States) were computed using the Robust Multichip Average algorithm [32]. In this study, we used a total of 480 microarrays: four animals × 30 RI strains × four tissues. Each individual tissue experiment consisted of 120 array datasets, which were processed and normalised separately. For fat, kidney, and adrenal tissues, cRNA was labelled and run on RAE 230A Affymetrix GeneChip arrays (number of transcripts 15,923). For LV tissue, cRNA was labelled and run on RAE 230_2 Affymetrix GeneChip arrays (number of transcripts 31,099).

Expression levels were generated and analysed independently for each tissue. The mean (SE) expression levels measured across 30 RI strains were 5.26 (0.01) for LV, 7.23 (0.02) for fat, 6.55 (0.01) for kidney, and 5.94 (0.01) for adrenal. The experimental variation within each tissue was evaluated through the coefficient of variation of gene expression among all transcripts represented in the microarrays. For all transcripts the average coefficient of variation (SE) was 3% (0.0001) for LV and 2% (0.0001) for fat, kidney, and adrenal tissues. The observed range of coefficients of variation was 0.3%–38% for LV, 0.4%–31% for fat, 0.3%–26% for kidney, and 0.8%–32% for adrenal. These estimates indicate that no significant differences were observed in the intra-specific variability of gene expression levels between tissues.

For this analysis we selected probesets that were mapped to the genome by Ensembl [33]. In order to avoid misclassification of *cis-* and *trans*-eQTLs, we also removed probesets that mapped to more than one place in the genome, resulting in 13,669 individual transcripts for fat, kidney, and adrenal and 27,168 individual transcripts for LV.

### Heritability of gene expression.

The narrow-sense *h* ^2^_trait_ in a set of RI lines was estimated using the method of Hegmann and Possidente [34], *h* ^2^_trait_ = 0.5*V*_A_/(0.5*V*_A_ + *V*_E_), where *V*_A_ represents the additive genetic component (variances of strain means) and *V*_E_ the average environmental component (variances within strains) [22]. Adjustments are usually required to correct for the fact that variance of strain means contains a proportion of the environmental component of variance. However, under conditions of reasonably large sample sizes from strains and relatively low variance/covariance within strains, this factor can be excluded from calculation. Because the correlation between the heritability estimates obtained using the complete and simplified formulas is very high (*r* ^2^ > 0.99), all estimates of *h* ^2^_trait_ discussed here were calculated using the simplified formula. Chi-square test for homogeneity and Spearman's correlations were calculated using SPSS 12.0 (SPSS, Chicago, Illinois, United States).

### Mapping of eQTLs.

We carried out genome-wide linkage analysis for each expression trait dataset generated in the RI strains in fat, kidney [11], adrenal, and LV tissues, and 1,011 genetic markers using the QTL Reaper program (http://sourceforge.net/projects/qtlreaper). For each transcript we calculated the likelihood ratio statistics for linkage and its empirical genome-wide significance [35] by permutations as previously described [11]. To account for multiple testing of thousands of expression traits, we calculated the FDR [36] and evaluated the expected number of falsely discovered eQTLs at each level of significance. The FDR of the *cis-* and *trans-*acting eQTLs was represented by vase box-plots [37], using the R package UsingR (http://www.math.csi.cuny.edu/UsingR) [38].

### Characterisation of eQTLs.

In this study, we empirically defined a *cis*-acting eQTL as having the peak of linkage within 10 Mbp of the physical location of the probeset and a *trans*-acting eQTL as having the peak of linkage on a different chromosome from where the probeset is located. Additive genetic effects (i.e., QTL allelic effects) and heritability of the eQTLs (*h* ^2^_QTL_) were calculated for each *cis-* and *trans*-eQTL. eQTL effects are an estimate of the absolute change in the transcript abundance that would be produced by substituting a single allele of one type with that of another type in the population. For each eQTL, its allelic effect is calculated as |MEAN_SHR_ − MEAN_BN_ |/2, where MEAN_SHR_ and MEAN_BN_ are the means of gene expression levels of all rats that respectively inherited SHR and BN alleles at the marker peak of linkage. The value *h* ^2^_QTL_ is the proportion of phenotypic variance that is attributable to the eQTL [39], and it is calculated from the squared allelic effect and the error variance segregating in the RI strains [40].

### Power calculation.

We calculated the expected statistical power to detect a major eQTL effect in the RI strains using the R package R/qtl Design (http://www.biostat.ucsf.edu/sen/software.html) [41]. In order to model various trait heritabilities, we considered different genetic and environmental variances within the experimental range observed in the RI strains across four tissues and used those estimates to calculate the power to detect a given additive effect of the eQTL [40]. The threshold used for detecting an eQTL was log of the odds score = 3.3 (i.e., genome-wide *p* = 0.05 [42]); 30 RI strains were considered. Similarly, we carried out additional power calculations and modelled the number of RI lines (30–200) and several thresholds of significance corresponding to those reported in this study.

### ROC curves.

For each transcript, the estimated *h* ^2^_trait_ was tested as a predictor of the presence of an eQTL (as detected by linkage) via ROC curve. ROC curves are plots of the true positive rate (sensitivity) of a diagnostic test against the test's false positive rate (1 − specificity) for various decision thresholds (i.e., various *p*-value cut-offs). The AUC measures the probability of correct classification, i.e., the ability of the trait heritability to correctly classify those eQTLs that will be (or will not be) detected in the linkage study. For a perfect prediction, the AUC will tend to be close to one and the ROC curve would follow the left-hand border and then the top border of the ROC space [25]. Non-parametric estimates of AUC and its SE were carried out using SPSS 12.0.

## Supporting Information

Figure S1 Comparison of Heritability of Gene Expression across Different Tissues Heritability of gene expression trait in the BXH/HXB panel of RI strains was calculated for each individual transcript considered in this study. The dotted line indicates the median heritability of gene expression. The histograms show similar distributions with a considerable heritability of gene expression in all tissues. The chi-square test did not show significant differences (*p* > 0.05 in all comparisons) between the distributions of heritability in different tissues.
(91 KB PPT)

Figure S2 Cumulative Frequency Curves of the Distribution of Transcripts Observed with a Given Heritability of Gene Expression in LV, Fat, Kidney, and Adrenal Tissues The relative proportions of heritable transcripts are similar in all tissues.
(46 KB PDF)

Figure S3 Comparison of ROC Curves for Prediction of Existence of a Detectable eQTL by Heritability of Gene Expression for LV, Fat, Kidney, and Adrenal Tissues Significantly detected eQTLs were defined at several levels of significance (*p*-value cut-offs 0.05, 10^−2^, 10^−3^, 10^−4^, and 10^−5^), and the relative ROC curves are compared. The vertical axis shows the fraction of major eQTLs that are correctly predicted (sensitivity) by the *h* ^2^_trait_, whereas the horizontal axis indicates the fraction of major eQTLs that are incorrectly predicted (1 − specificity) by the estimate of the *h* ^2^_trait_. The closer the ROC curve is to the upper left-hand corner of the graph, the more accurate is the prediction of the eQTL (i.e., higher true positive rate and lower false positive rate). The areas under the ROC curves are reported in Table S1.
(3.5 MB PPT)

Figure S4 Statistical Power to Detect an eQTL with Allelic Effects 0.06, 0.1, 0.15, and 0.2 (FC 1.09, 1.15, 1.23, and 1.32) Various estimates of heritability of gene expression were modelled, and 30 RI strains were considered (see Materials and Methods).
(67 KB PPT)

Figure S5 Statistical Power to Detect an eQTL with Allelic Effect 0.1 (i.e., FC 1.15) as a Function of the Number of RI Strains Various estimates of heritability of gene expression were modelled, and four biological replicates were considered.
(39 KB PPT)

Figure S6 Cumulative Frequency Curves of the Distribution of *Trans*-eQTLs Detected with Genome-Wide Significance (*p* = 0.05) with a Given Allelic Effect in LV, Fat, Kidney, and Adrenal Tissues We observe an enrichment of *trans*-eQTLs with small allelic effects in the range 0–0.8 for kidney and adrenal tissues compared with LV and fat. Insert: cumulative frequency curves of the distribution of *trans*-eQTLs for allelic effects are in the range 0–0.16.
(57 KB PPT)

Table S1 AUCs for Prediction of Existence of a Detectable eQTL by Heritability of Gene Expression for LV, Fat, Kidney, and Adrenal Tissues (46 KB DOC)

## Abbreviations

AUC: area under the receiver operating characteristic curve
BN: Brown Norway
eQTL: expression quantitative trait locus
FC: fold change
FDR: false discovery rate
*h^2^*_QTL_: heritability of the QTL
*h^2^*_trait_: trait heritability
LV: left ventricle
QTL: quantitative trait locus
RI: recombinant inbred
ROC: receiver operating characteristic
SE: standard error
SHR: spontaneously hypertensive rat
