# Supplementary material for: Heritability and Tissue Specificity of Expression Quantitative Trait Loci
Source: PLoS Genet. 2006 Oct 20;2(10):e172. doi: 10.1371/journal.pgen.0020172 (PMC1617131; doi:10.1371/journal.pgen.0020172)
Supplement: Supplementary file 1 [file pgen.0020172.sg002.pdf]

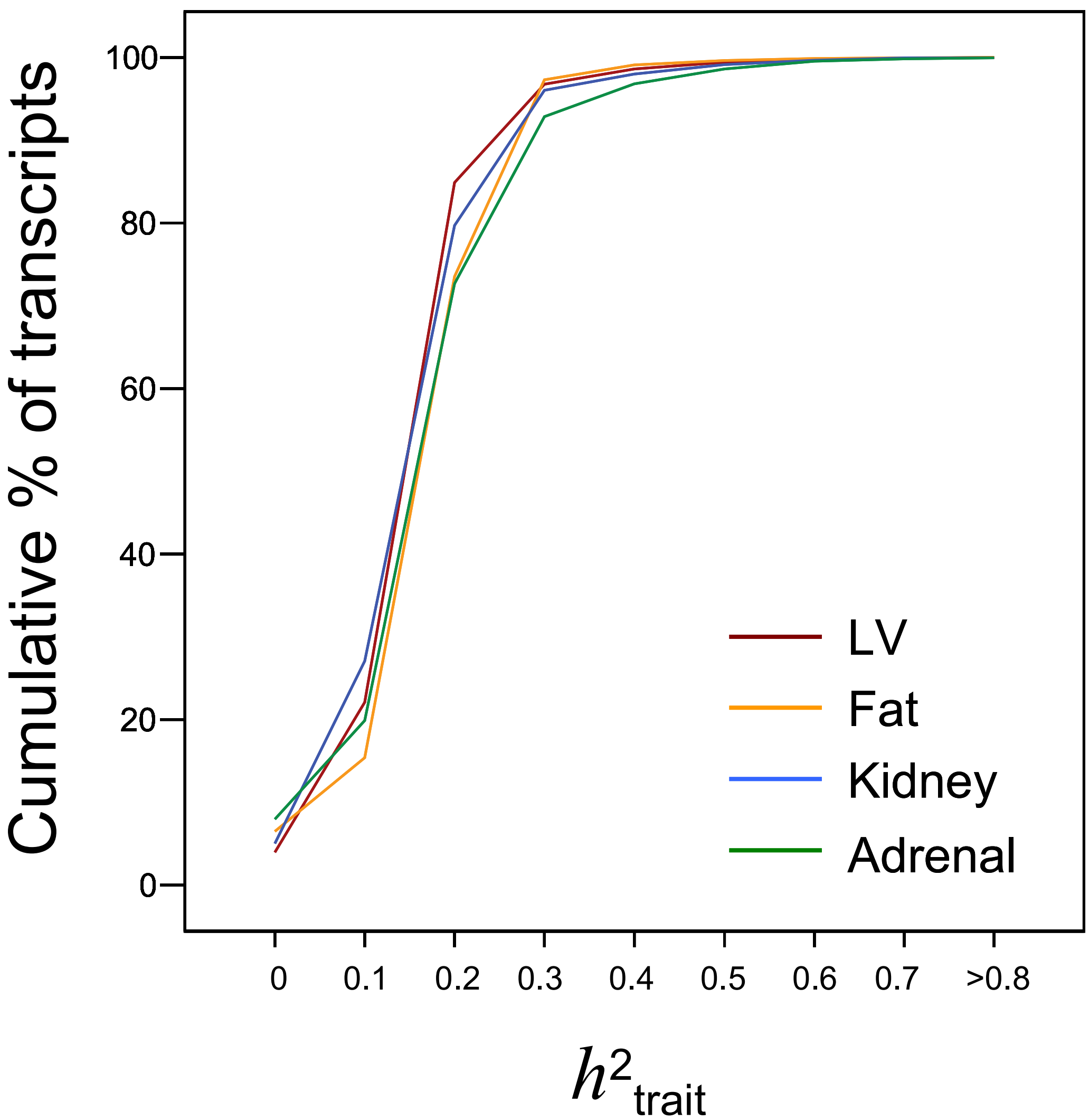

Cumulative % of transcripts
100
80
60
40
20
0
0
0.1
0.2
0.3
0.4
0.5
0.6
0.7
>0.8
h^2_{trait}
LV
Fat
Kidney
Adrenal
