# Supplementary material for: Heritability and Tissue Specificity of Expression Quantitative Trait Loci
Source: PLoS Genet. 2006 Oct 20;2(10):e172. doi: 10.1371/journal.pgen.0020172 (PMC1617131; doi:10.1371/journal.pgen.0020172)
Supplement: Supplementary file 2 [file pgen.0020172.st001.doc]

|  | *Area under the ROC curve (SE)* | | | |
| --- | --- | --- | --- | --- |
| *p*-value cut-off for detection of the eQTL | LV | Fat | Kidney | Adrenal |
| 0.05 | 0.71 (0.005) | 0.68 (0.007) | 0.73 (0.007) | 0.68 (0.008) |
| 0.01 | 0.82 (0.007) | 0.78 (0.012) | 0.82 (0.009) | 0.77 (0.011) |
| 0.001 | 0.91 (0.007) | 0.93 (0.009) | 0.90 (0.008) | 0.88 (0.011) |
| 0.0001 | 0.95 (0.006) | 0.95 (0.011) | 0.95 (0.006) | 0.94 (0.011) |
| 0.00001 | 0.97 (0.004) | 0.97 (0.011) | 0.95 (0.009) | 0.94 (0.016) |
